# Prediction of HIV-1 sensitivity to broadly neutralizing antibodies shows a trend towards resistance over time

**DOI:** 10.1371/journal.pcbi.1005789

**Published:** 2017-10-24

**Authors:** Anna Hake, Nico Pfeifer

**Affiliations:** 1 Department Computational Biology and Applied Algorithmics, Max Planck Institute for Informatics, Saarland Informatics Campus, Saarbrücken, Germany; 2 Methods in Medical Informatics, Department of Computer Science, University of Tübingen, Germany; 3 Medical Faculty, University of Tübingen, Tübingen, Germany; Helmholtz-Zentrum fur Infektionsforschung GmbH, GERMANY

## Abstract

Treatment with broadly neutralizing antibodies (bNAbs) has proven effective against HIV-1 infections in humanized mice, non-human primates, and humans. Due to the high mutation rate of HIV-1, resistance testing of the patient’s viral strains to the bNAbs is still inevitable. So far, bNAb resistance can only be tested in expensive and time-consuming neutralization experiments. Here, we introduce well-performing computational models that predict the neutralization response of HIV-1 to bNAbs given only the envelope sequence of the virus. Using non-linear support vector machines based on a string kernel, the models learnt even the important binding sites of bNAbs with more complex epitopes, i.e., the CD4 binding site targeting bNAbs, proving thereby the biological relevance of the models. To increase the interpretability of the models, we additionally provide a new kind of motif logo for each query sequence, visualizing those residues of the test sequence that influenced the prediction outcome the most. Moreover, we predicted the neutralization sensitivity of around 34,000 HIV-1 samples from different time points to a broad range of bNAbs, enabling the first analysis of HIV resistance to bNAbs on a global scale. The analysis showed for many of the bNAbs a trend towards antibody resistance over time, which had previously only been discovered for a small non-representative subset of the global HIV-1 population.

## Author summary

Several sequence-based approaches exist to predict the epitope of broadly neutralizing antibodies (bNAbs) against HIV based on the correlation between variation in the viral sequence and neutralization response to the antibody. Though the potential epitope sites can be used to predict the neutralization response, the methods are not optimized for the task, using additional structural information, additional preselection steps to identify the epitope sites, and assuming independence and/or only linear relationship between the potential sites and the neutralization response. To model also the neutralization response to bNAbs with more complex binding sites, including for example several non-consecutive residues or accompanying conformational changes, we used non-linear, multivariate machine learning techniques. Though we used only the viral sequence information, the models learnt the corresponding binding sites of the bNAbs. In general only few residues were learnt to be responsible for a change in neutralization response, which can additionally reduce the sequencing cost for application in clinical routine. We propose our tailored models to aid the patient selection process for current clinical trials for bNAb immunotherapy, but also as a basis to predict the best combinations of bNAbs, which will be required for routine clinical practice in the future.

## Introduction

With around 36.7 million people living with HIV in 2015 and an incidence rate of around 2.1 million each year [1], infections with HIV continue to be a major global health issue. However, despite more than three decades of research, there is neither a vaccine against nor a cure available for infection with HIV-1. HIV-1 infected patients are usually treated with a highly active antiretroviral therapy (ART). ART suppresses the replication of the active virus, but it is not capable of eliminating viral reservoirs and thus clearing the infection. To reduce the emergence of drug-resistant viruses, ART usually consists of a combination of three or more drugs from at least two different drug classes. In total, there are six different drug classes, which differ in their mode of interference with the HIV-1 life cycle, resulting in more than 20 available antiretroviral drugs. A change of the drug regimen is still often required, due to emerging drug resistances or side-effects. Since lifelong treatment is inevitable, for some patients no efficient drug regimens might be left eventually. Hence, there is still a high demand for drugs with new targets [2].

A currently investigated treatment option is the passive transfer of a combination of broadly neutralizing antibodies (bNAbs) to HIV-1 patients. Upon the advent of new single-cell antibody cloning techniques [3–5] and followed structure-based rational design approaches [6], an abundance of these new bNAbs has been isolated and their higher neutralization potency and breadth have been shown in several studies [6–10]. The potency of an antibody is defined as the antibody concentration needed to inhibit HIV-1 infectivity by 50% (IC50) or 80% (IC80), while the neutralization breadth of an antibody is measured by the ability of the antibody to neutralize viruses from different subtypes. The latter characteristic is very important in the case of HIV-1 due to its high molecular diversity within a patient but also within a population.

The sole target of these neutralizing antibodies is the viral envelope glycoprotein, the so-called envelope spike, on the surface of the virus. The surface of the virus itself is made of host-lipids and is therefore undetectable by the immune system. Each spike consists of a trimeric heterodimer of two viral envelope glycoproteins, gp120 and gp41, which are cleaved from the envelope glycoprotein, gp160. While gp41 mediates host cell fusion, gp120 is essential for cell entry [11]. By successful binding of a neutralizing antibody to a spike, a chain reaction is initiated by the host immune system that eventually leads to the elimination of the virus.

So far, there are five known sites on the envelope glycoprotein, which are targeted by a variety of bNAbs (given in brackets): on gp120 the CD4 binding site (e.g., VRC01, VRC-PG04, 3BNC117, NIH45-46) [9, 12–14], the V1/V2 region (e.g., PG9 and PG16) [7, 8, 15–17], and the V3 loop (e.g., PGT128, PGT121, 10-996, 10-1074) [8, 10, 18–21]; the membrane proximal external region (MPER) on gp41 (e.g., 10E8) [22–25]; and a newly identified site comprising parts of gp41 and gp120 (e.g., 35O22) [26]. Since the specific binding sites of bNAbs, so-called epitopes, on the envelope protein are not similarly accessed by any available drug, a therapy with bNAbs would offer a new effective treatment option for patients with resistance to all current therapies or might boost existing therapy combinations with few active drugs [27]. The efficacy of a treatment with a combination of these broad and potent neutralizing antibodies has been first shown in HIV-1 infected humanized mice [28, 29] and non-human primates [30]. Tolerance and safety of the bNAbs VRC01 [31] and 3BNC117 [32] have been shown in phase 1 clinical trials in HIV-1 infected humans, where for 3BNC117 also the effective suppression of viremia could be observed. In addition, recent studies have shown that antiretroviral therapy with only one bNAb (3BNC117) is able to enhance the host immune response against HIV-1 [33] and leads to a significant delay of viral rebound after treatment interruption [34]. In contrast to ART, which usually requires a daily intake of the drugs, bNAbs have a longer half-life time, being able to control the viral load for more than 28 days in humans after administration [32]. High genetic variation of the viral envelope glycoproteins together with a glycan shielding of more conserved regions on the envelope often allow the virus to escape immune recognition [35]. Thus, for treatment success, neutralization resistances of the patient’s viral strains to the given bNAbs must be detected beforehand. Up to now, the neutralization sensitivity of a virus to an antibody can only be determined in time-consuming and expensive neutralization assays.

To ensure a routine clinical practice, these tests have to be more rapid and cost-effective. This can be achieved, for example, by developing a genetic resistance test, coupled with a resistance prediction method similar to current decision support for ART treatment against HIV [36]. Since the envelope spike is the sole target of bNAbs, it is sufficient to consider the changes in the genetic composition of the viral envelope glycoproteins associated with changes in neutralization sensitivity of the virus.

So far, the neutralization together with the genetic information has been mainly used to determine potential epitopes of bNAbs or to identify immunogens to elicit bNAbs. The aim of neutralization-based epitope prediction models is to learn potential epitopes or patches of the bNAb in the amino acid sequence of the envelope protein. There are approaches using only the neutralization information [37–41] or including structural information [41, 42]. Changes in the amino acid composition of the epitopes are assumed to be associated with a change in neutralization sensitivity and thus can be learned from neutralization activity information. As a consequence, the model learns potential sites instead of predicting neutralization sensitivity. Nevertheless, some of the models, or more precisely the learnt sites, have been used to predict the neutralization activity for validation purpose. Unfortunately, the performance might be overoptimistic if the same data is used for learning the sites and the prediction task [42]. Another application is the identification of immunogens to elicit bNAbs. Therefore, Gnanakaran et al [43] compared the viral sequences of HIV-1 infected individuals with and without a broad and potent antibody response, hypothesizing that shared features among the viral sequences in individuals eliciting bNAbs might be potential immunogens. Shared features have been learnt using conditional mutual information together with an ensemble learning technique using classification trees. Similar to the above approaches, the identified features have been validated by predicting the neutralization sensitivity. An overview of a variety of computational approaches for epitope vaccine design is given by He et al. [44].

Recently, an artificial neural network approach has been proposed to directly model the IC50 value based on the envelope sequence information [45]. For this, the amino acids were mapped to integers. However, the authors modeled each position in the sequences as a continuous variable instead of a categorical one, which leads to a different interpretation of changes between different amino acids. In addition, only the performance of the older bNAb 2F5 was provided. IDEPI [46] is a very generic framework that, among other features, models the neutralization sensitivity of the virus to bNAbs using a linear support vector machine (SVM) and the envelope sequence of the virus. The above presented models have several shortcomings. First, potential epitopes can be poor immunogens. Second, sites outside the epitope can have an influence on the binding success of a bNAb as well, and thus also have an influence on the neutralization sensitivity. Structural information and other prior information about the binding sites might not be available for newly identified bNAbs. Most methods assume a linear relationship between changes in the amino acid composition and neutralization sensitivity on the one hand [37, 46] and the independence of the epitope sites on the other [37]. This assumption might not hold for bNAbs targeting a more complex binding site. Another important point involves the handling of amino acid positions in the variable regions of the envelope protein. Though the variable regions are hard to align, they are also the regions where resistance mutations are likely to appear and thus these sites should not be dropped from the analysis [43].

In this study, we present prediction models for 11 different bNAbs (VRC01, VRC-PG04, 3BNC117, NIH45-46, PG9, PG16, PGT121, PGT128, 10-996, 10-1074, and 35O22) that learnt discriminant *signals* (amino acids or patterns of amino acids) in the genetic sequence of the envelope glycoprotein gp160 (*envelope sequence*), which influence the neutralization sensitivity to the particular antibody. To learn the neutralization susceptibility of HIV-1 strains to bNAbs, we trained our prediction models on data from three previously published neutralization assays [10, 26, 47]. Depending on the neutralization assay, IC50 titers for 115 to 220 HIV-1 isolates were available for each of the bNAbs. Following neutralization assay protocols, we used an IC50 value above 50 μg/mL as a threshold to determine neutralization resistance of a virus to a particular antibody. Based on the available IC50 titers for the HIV-1 isolates, the corresponding envelope sequences, and the threshold, we built binary classifiers with non-linear support vector machines (SVM) and string kernels to distinguish between HIV-1 resistance and susceptibility to a bNAb. As non-linear prediction models are often seen as black boxes, we trace back what each classifier learnt from the data and show that many of the learnt discriminant signals are known to play an important role for the binding success of the antibody. For a better interpretation of the classification decision (resistant or susceptible), we provide a new way to produce motif logos that illustrate which and up to what extent amino acids in the tested sequence contributed to the particular classification result. Though we use the complete envelope sequence information, we show that only a few signals are important for the classification outcome and that models based only on these signals achieve comparable prediction power.

To study the evolution of HIV-1 resistance to bNAbs, we additionally built regression models using support vector regression that directly predict the IC50 value from the envelope sequence of the virus. With these models we analyzed the neutralization sensitivity of HIV-1 to the considered 11 bNAbs for around 34,000 HIV-1 samples of different subtypes over a time period of more than 30 years from the Los Alamos HIV sequence database [48]. Thereby, we could not only confirm previous, experimental results, showing that there is a trend towards bNAb HIV-1 resistance over time in the subtype B population of HIV-1 on a much larger and more diverse data set, but for the first time, the trend could also be observed for the global HIV-1 population—a scale-up that would be very expensive in an experimental setting.

A preliminary version of this study [49] has been published as a preprint.

## Results and discussion

### Prediction performance

#### Accurate prediction of bNAb resistance from the genetic sequence of the envelope protein of HIV-1

**Fig 1:**
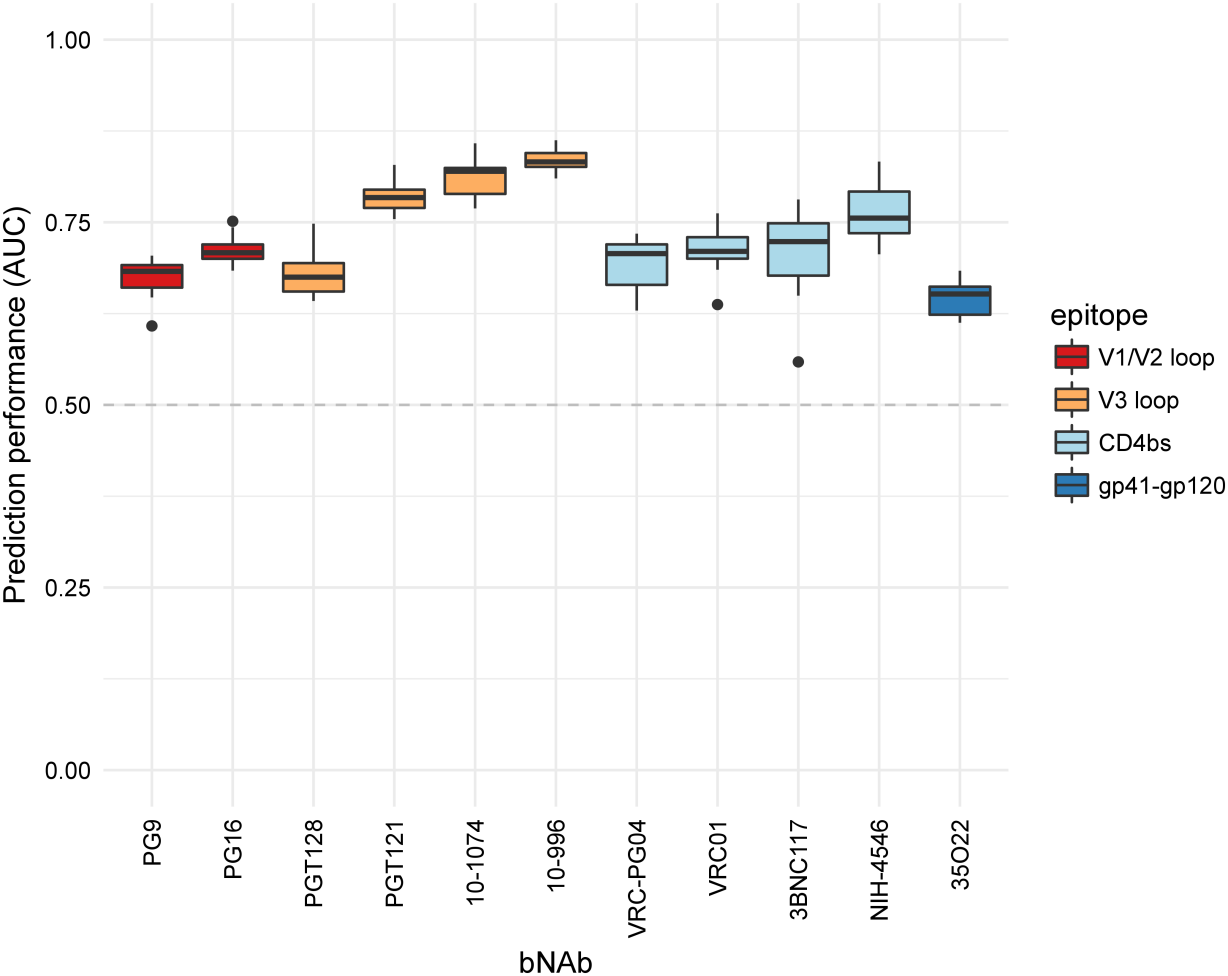
Prediction performance of the classifiers. The AUC performances for each bNAb classifier using the oligo kernel were determined by 10 runs of a stratified 5-fold nested cross-validation. On the x-axis, the different classifiers are presented, named according to the bNAb they are trained on. The colors of the boxes refer to the epitope category of the corresponding bNAb. The prediction performance of a random classifier is depicted by the gray dashed line.

We used support vector machine (SVM) models to build our prediction models. A crucial step in building SVM models is the choice of the kernel that encodes the similarity structure in the input data. Upon performance comparison between different kernels (see S1 Table), the oligo kernel was selected for all bNAbs to predict the neutralization susceptibility to each bNAb for new viral strains. The idea of the oligo kernel is to define the similarity between two sequences *x* and *x*′ of same length *L* by the similarity of the co-occurrences of their substrings (oligomers) of length *l* with 1 ≤ *l* ≤ *L* within a certain distance (controlled by the width parameter σ^2^). Fig 1 shows the prediction performance of each of the 11 classifiers measured as the area under the ROC curve (AUC). The prediction performance was assessed in 10 runs of a stratified 5-fold nested cross-validation in the kernel comparison step. All 11 classifiers are better than a random classifier (dashed line) and have good performances, up to 0.84 AUC for the V3 loop targeting bNAbs. The prediction performances of the regression models are provided in S1 Fig. To determine the best parameter setting for each bNAb prediction model, we performed an additional 5-fold cross-validation.

#### Comparison to other machine learning approaches

Due to the large number of features (the length of the envelope amino acid sequence) compared to the small number of samples, we chose SVMs to build our models, which are known to generalize well for these kind of prediction problems. Additionally, we compared our final SVM models (based on the oligo kernel) to a selection of other machine learning approaches: random forests, SVM using a linear kernel, a neural network, and a logistic regression with lasso regularization (see Methods for details). Overall, only the random forest approach and our model performed well for all 11 bNAbs while not being significantly different performance wise. Similar to the other investigated kernels, the linear kernel had worse performance for the VRC-PG04 bNAb compared to the oligo kernel or the random forest approach. The prediction performances are presented in S6 Fig and S5 Table. There are a plethora of machine learning approaches that could be used to tackle the here discussed classification task. Thus, we do not claim that there cannot be a better method than SVMs based on the oligo kernel. From our analysis, it seems that for bNAbs that need a single specific amino acid for a successful binding such as the V3-loop or V1/V2-loop targeting bNAbs, simpler models will perform equally well as the oligo kernel approach. Depending on the learnt hyperparameters, the oligo kernel however can also capture more complex l-mers, an advantage if the binding site pattern of the bNAb is not known beforehand.

### Model reliability and user features

#### Learnt hyperparameter of prediction models agree with binding patterns of bNAbs

Table 1 presents the final parameters settings for the classifiers for the bNAbs PG9, PG16, 10-669, 10-1074, PGT121, VRC01, and VRC-PG04 fitted by a stratified 5-fold cross-validation. For the PGT121 and VRC-PG04 classifier an *l*-mer of length 6 led to the best performance whereas the *l*-mer length for the other antibodies was comparatively small (2-mers for VRC01 and single positions for the remaining antibodies). The length differences of the *l*-mers for different epitope classes supports the knowledge gained from experimental findings. For the N-glycan dependent antibodies, a single glycan site is the most important residue for successful binding. The N332-linked (V3 loop directed) antibodies PGT121, 10-1074, and 10-996 need in the first instance an asparagine at position 332 for successful binding [16]. The N160-linked antibodies PG9 and PG16 bind in a hammerhead-like way to the virus, building contacts with two glycans (160 and 156 or 171) [15]. For the CD4 binding site (CD4bs), which forms a cavity, it is only known that it is sterically not easy to bind to for antibodies [50]. Longer *l*-mers led to the best prediction results for the CD4bs classifiers, which is likely due to the fact that the CD4bs-directed bNAbs target a larger epitope compared to the other bNAbs.

**Table 1:** Final parameter settings for the oligo kernel classifiers for each bNAb.

Epitope	bNAb	l	width
V1/V2 Loop	PG9	1	1
	PG16	1	0.4
V3 Loop	PGT121	6	1.6
	10-996	1	2.6
	10-1074	1	1.6
CD4bs	VRC01	2	3.6
	VRC-PG04	6	20

The parameter *l* denotes the size of the *l*-mer and the parameter *width* (σ^2^) the allowed positional uncertainty of the kernel.

#### Classifiers learnt important binding sites

In general, the learnt signals of a non-linear kernel-based SVM classifier can be traced back, if the kernel incorporates positional information such as the weighted degree kernel with shifts (WDKS) [51] or the oligo kernel [52]. By construction of the oligo kernel (see Methods), it is possible to retrieve the learnt weight of all occurring oligomers at each position in the sequence to the classifier.

Considering the 15% strongest learnt signals for each classifier, we found that several amino acids (*residues*) of the envelope protein were learnt by the classifiers to influence neutralization resistance or susceptibility, which are also supported by literature [39, 53]. In Table 2 we present the learnt signals of the classifiers exemplarily for the bNAbs PG9, PG16, 10-669, 10-1074, PGT121, VRC01, and VRC-PG04 that are supported by previous studies.

**Table 2:** Learnt discriminant signals by each bNAb classifier that are supported by literature.

bNAb	susceptible	resistant
PG9	N160, N301, S393, S613, K168, K169, K171	N624, D187
PG16	N136, N141, N160, N186, N234, N289, N356 S393, K169, K171, D167, T138	N230
VRC01	N186, N276, N279, N280, G459, K232	
VRC-PG04	N186, N276, N279, N280, G459, K232, R456, D368	
10-996	N332, S334	N334
10-1074	N332, S334	N334, T388, T818
PGT121	QAHCN328-332, R332	

Signals among the 15% strongest learnt signals for each classifier were considered.

Most of the found discriminant signals for the N-glycan dependent antibodies, that is, for the V1/V2 loop and V3 loop directed antibodies, contain the amino acids asparagine (N), serine (S) and threonine (T). These amino acids are also part of the pattern N-X-[S or T], which defines potential N-glycosylation sites [54]. The classifiers for the CD4bs antibodies identified known required residues for CD4-binding as reported in [53]. The fact that all classifiers learnt some known discriminant position, further support the reliability of the prediction models in addition to the provided prediction performances. Additionally to the already known epitope sites, we found further discriminant residues whose role needs to be validated in knock-out experiments and might be interesting for follow-up structural studies (see S7 Table for a complete list of 1% discriminant signals).

#### Motif logo improves classifier interpretability

To improve the interpretability of the classification decision, we show how to produce for each classification of a test envelope sequence a motif logo—a representation of the test sequence—that displays those residues in the test sequence that contributed the most to the classification result. Using the available kernel feature representation of the oligo kernel, it is possible to retrieve the contribution of each residue of the test sequence to the classification. As the envelope glycoprotein consists of around 800 amino acids, visualizing the contribution of all amino acids to the classification would not be very informative. Instead, since the prediction performance of classifiers based only on the strongest p% signals with p ∈ {1, 3, 5, 7, 10, 15, 20, 25} performed not significantly worse than the classifiers based on the complete envelope sequences (see S4 Table), we present only the contribution of the strongest signals in the motif logo.

**Fig 2:**
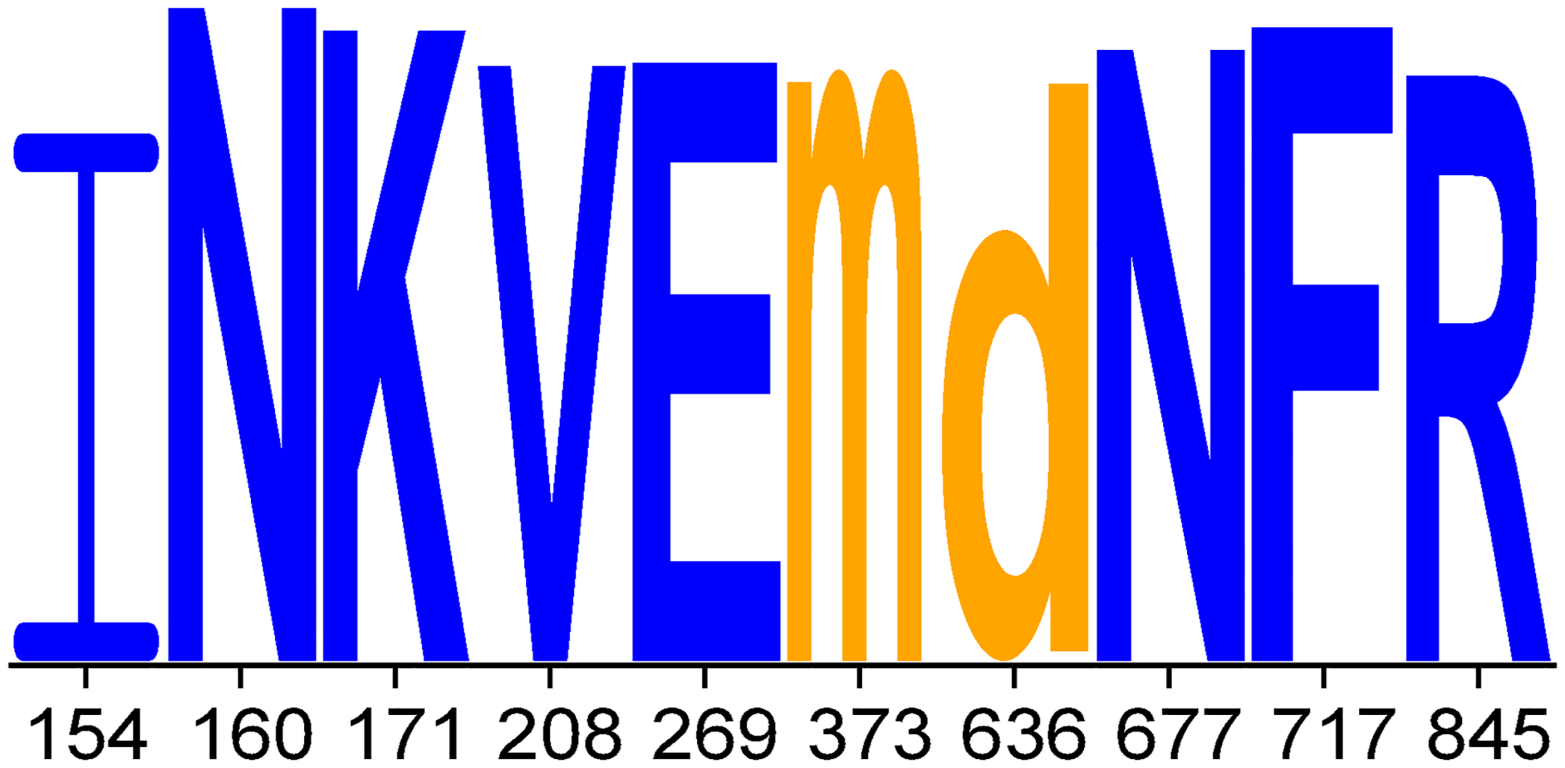
Motif logo for the test sequence HM469973 using the PG9 classifier. For the motif logo the contribution of 5% of the strongest discriminant signals to the classification is considered. The height of the letters depends on the proportional contribution to the classification. Amino acids of the test sequence that influence the classification outcome towards neutralization susceptibility are displayed in capital letters and blue color; lowercase letters and orange color if they contribute to neutralization resistance. For better interpretability, the corresponding positions of the amino acids in the envelope sequence of the HIV strain HXB2 are shown on the x-axis.

For demonstration purposes, we retrieved several HIV-1 envelope sequences from the Los Alamos HIV sequence database [48] serving as test input for the classifiers. In Fig 2 we present the motif logo for the test sequence with the GenBank ID HM469973, which was classified by the PG9 classifier as susceptible, using the strongest 5% learnt discriminant signals to the classification outcome of the test sequence. The asparagine (N) at position 160, which is known to be decisive for a successful binding of the PG9 bNAb, as well as the lysine (K) at position 157 have the highest contribution to the classification result, more precisely to susceptibility. In general, most of the 5% strongest signals influence the classification result towards susceptibility.

### Trend towards bNAb resistance over time

In order to investigate whether neutralization sensitivity of HIV-1 to bNAbs has changed over time, we additionally built support vector regression models to directly predict the (logarithmized) IC50 value for the 11 considered bNAb. For subtype B variants, a continuous trend towards resistance has been already confirmed in certain cohorts (around 40 samples) of the French and Dutch HIV-1 population [55–57]. Since evolving resistance to antibody neutralization in the HIV-1 species would have major implications on the antibody selection for current vaccine development, it is important to know whether such a drift towards resistance also exists in the global HIV-1 population for all subtypes. In contrast to an experimental setting, where the large number of viral strains and the accruing costs make neutralization assays for the comprehensive global population hardly possible, our prediction models can be easily used to examine this question based on the vast amount of available sequence data.

To model the global HIV-1 population over time, we used all available envelope sequences from the Los Alamos HIV sequence database (around 34,000 after data processing, see Methods and S9 Table for accession numbers) comprising viral isolates from all major subtypes over a time interval from 1981 to 2013. We divided the given time interval into the following six time periods to account for changes in HIV-1 treatment strategies: 1981-1986 before ART, 1987-1991 ART monotherapy, 1992-1995 ART combination therapy (cART), 1996-1999 cART with protease inhibitors, 2000-2005 cART with Lopinavir/Ritonavir, and 2006-2013 cART with Maraviroc/Raltegravir. With this partitioning of the data, we additionally covered the considered time intervals in the previously performed experimental studies [55–57]. An overview of the different subtypes and country distribution per time period are displayed in S2 Fig.

In order to identify a drift towards resistance, we performed a permutation test for umbrella alternatives [58] on the predicted (logarithmized) IC50 values grouped by the six time periods. The umbrella test [59] is a more general test than the Jonckheere-Terpstra test [60, 61]. Instead of testing for a monotonic trend, it tests for a peak in one of the time periods—a trend, monotonically increasing before and decreasing after the peak. The permutation test of umbrella alternatives [62] provides in additional partial p-values for each group, which enables a better analysis of the trend. Here, we define a trend towards resistance, if the peak is in the last time periods (see Methods for details). In contrast to the experimental studies [55–57], our data set is much larger, covers longer time periods, and is more heterogeneous. Thus, we expected to see more variation in our groups and therefore decided to use the umbrella test as a more general test in our case. However, we additionally provide the statistics for the Jonckheere-Terpstra test in S6 Table, which can be seen as a more conservative test.

When considering only the subtype B variants of the around 34,000 viral isolates (17,392), we observed a statistically significant increase of the predicted (logarithmized) IC50 values over the six time periods to each of the 11 bNAbs (*P* ≤ 0.001 using the umbrella test and a significance threshold *t* = *α*/#*tests* = 0.05/22 = 0.0023 with Bonferroni correction for multiple testing). Thus, we could confirm the trend towards bNAb resistance [55–57] on a larger and more diverse data set. The predicted (logarithmized) IC50 values for the subtype B samples for all 11 bNAbs are provided in S3 Fig. Note that in order to avoid misleading data visualization, we present all the predicted values for all 11 bNAbs on the same y-scale, though the bNAbs differ in their neutralization strength. Though we find the last time periods as part of a significant trend in the data for PG9, PG16 and PG128, the partial p-values indicate rather a plateau distribution than a clear trend towards resistance in the last time periods (see S6 Table).

**Fig 3:**
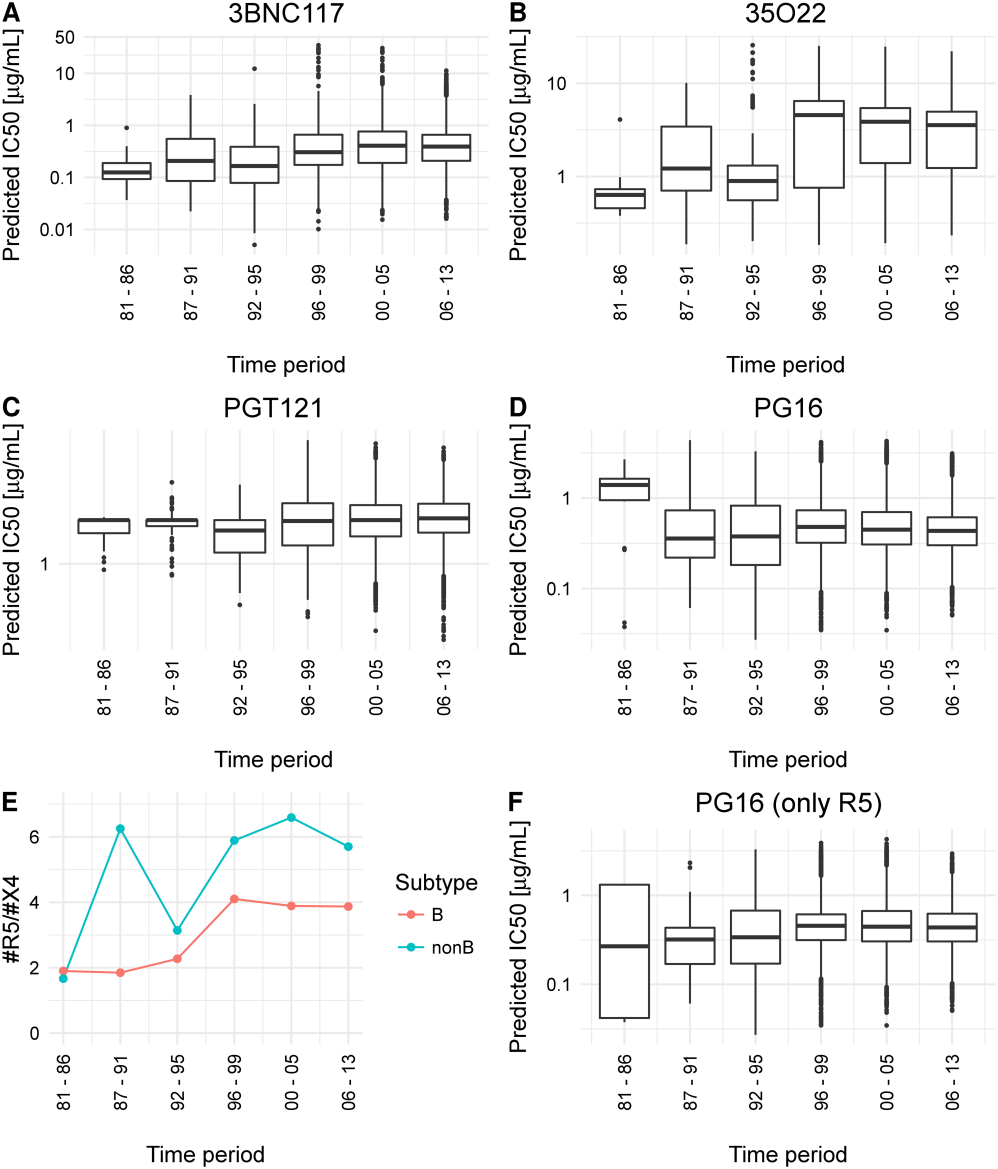
Characteristics of the non-B subtype HIV variants over time. Predicted neutralization sensitivity of non-B subtype HIV-1 variants to bNAbs over time. **A** - **D**: the predicted neutralization sensitivity of HIV-1 samples of the non-B subtype to the bNAbs 3BNC117, 35O22, PGT121, and PG16. **E**: the ratio of R5-tropic and X4-capable non-B subtype HIV-1 variants in the Los Alamos HIV sequence database over the six time periods. **F**: predicted neutralization sensitivity of R5-tropic variants of the non-B subtype to PG16.

**Fig 4:**
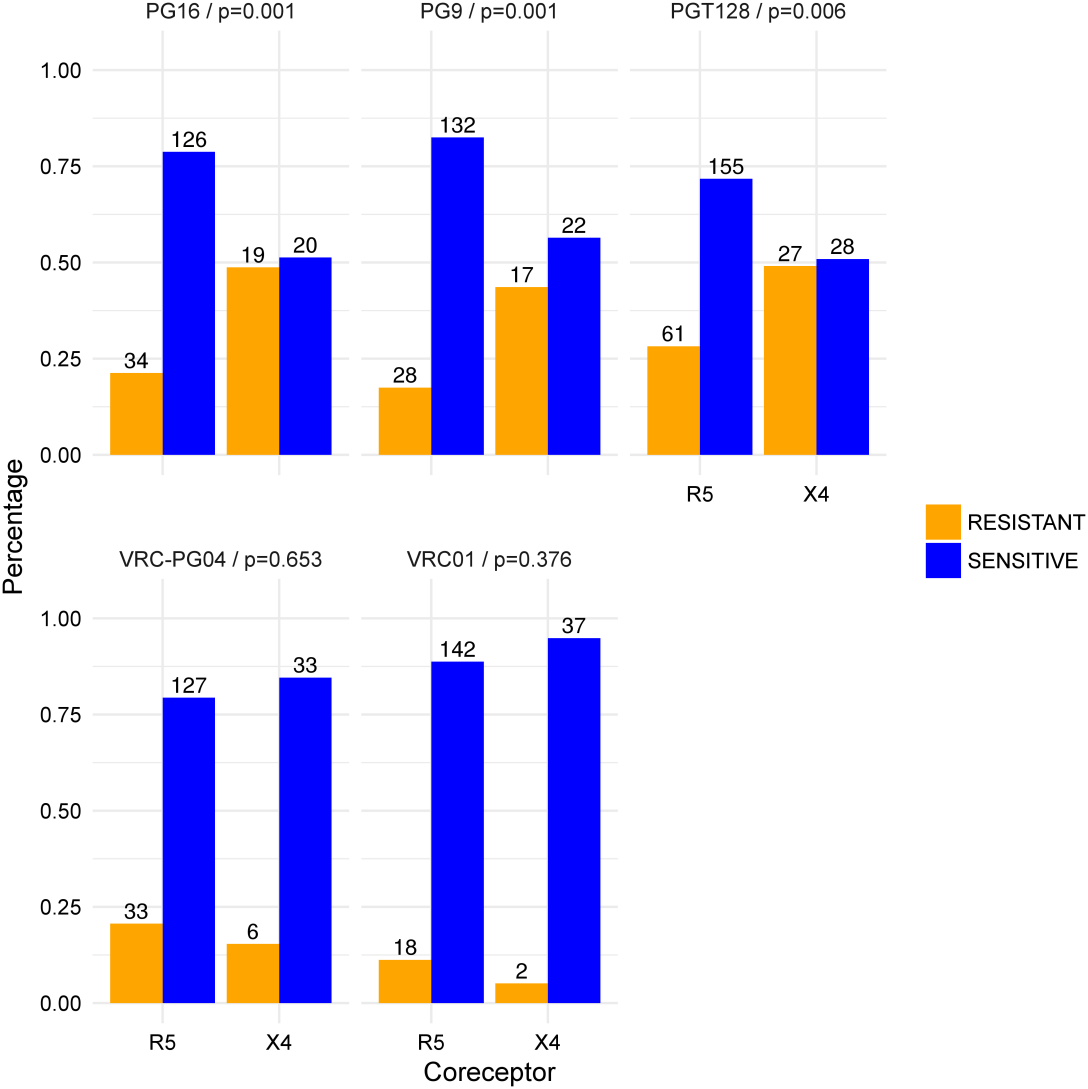
Association between coreceptor usage and neutralization sensitivity. Relative number of resistant (orange) and susceptible (blue) strains with regard to their coreceptor usage for the bNAbs PGT128 and VRC-PG04, as well as for VRC01, PG9, and PG16. Statistical significance was assessed with a two-sided Fisher’s exact test. Data for VRC01, VRC-PG04, PG9 and PG16 was taken from Pfeifer et al. [63].

In addition, we predicted and analyzed the neutralization sensitivity of the non-B subtype samples (16,546) to the 11 bNAbs. A statistically significant trend towards resistance was observed for all considered bNAbs, but PG9, PG16, PG121,PGT128 and NIH-4546. In Fig 3 we show exemplarily the predicted (logarithmized) values for the bNAbs (A) 3BNC117 (CD4bs), (B) PGT121 (V3 loop), (C) 35O22 (gp41/gp120), and (D) PG16 (V1/V2 loop); see S4 Fig for all bNAbs and non-B subtype samples. While for the bNAb PGT128 there was no significant peak at all, the trend towards resistance to the bNAb PGT121 was not significant after Bonferroni correction for multiple testing. For PG9, PG16 and NIH-4546, we detected a significant peak in the data, but not in the last time period, which we however required to determine a trend towards resistance (see Methods for details). The peak for NIH-4546 was slightly shifted (in the fifth time period), whereas for PG9 and PG16 a significant peak was already detected in the first time period, that is, the HIV variants tend to become more susceptible in the last time period. Since there are no experimental data on HIV-1 resistance development trends to bNAbs for the non-B subtype population, we decided to first rule out the possibility of a confounder that might lead to the contrasting trend for PG9 and PG16. Pfeifer et al. [63] discovered that there is a statistically significant bias in the neutralization susceptibility of HIV-1 variants to PG9 and PG16 depending on the coreceptor usage of the virus. For successful entry of the virus into the host cell, the glycoprotein gp120 has not only to bind to the CD4-receptor on the host cell, but also to a second chemokine receptor on the host cell that acts as co-factor (coreceptor). The coreceptors mainly used by HIV-1 are CCR5 and CXCR4. Depending on the coreceptor usage, the virus strain is referred to as R5- or X4-tropic, or dual-tropic if the virus can bind to both of these coreceptors, and X4-capable, if they are either dual-tropic or X4-tropic [64]. X4-capable viruses have been shown to be more resistant to PG9 and PG16 [63]. This means that PG9 and PG16 have an R5-bias, that is, they are better in neutralizing R5-tropic viruses than X4-capable viruses. By determining the coreceptor usage for all considered viral samples with the most widely used tool for genetic tropism testing, geno2pheno[coreceptor] [65], we detected a stronger increasing ratio of R5- to X4-capable viruses over the time periods for the non-B than for the subtype B samples (see Fig 3E and S2 Table). Thus, we might see an increase in neutralization susceptibility to PG9 and PG16 due to the relative increase of R5-tropic variants in the later time periods, since R5-tropic variants are more susceptible to PG9 and PG16. With an analysis, analogous to Pfeifer et al. [63], we observed an R5-bias of the bNAb PGT128 (*P* = 0.00568 using a two-sided Fisher’s exact test, see also S3 Table). Fig 4 shows the relative number of resistant and susceptible HIV strains to PGT128 in comparison to PG9, PG16, VRC-PG04 and VRC01. Data for VRC01, VRC-PG04, PG9 and PG16 was taken from Pfeifer et al. [63]. We additionally analyzed the association between coreceptor usage and neutralization sensitivity for all considered 11 bNAbs. As can be seen in S5 Fig, we could not detect other bNAbs with an R5-bias. For the bNAb PG16, a resistance trend was only detected for the R5-tropic variants (see Fig 3F). Note that sequences from the beginning of the HIV epidemic (first two time periods) were probably from patients having AIDS and not at early stage of HIV infection as nowadays. Since at early stage of clinical HIV infection usually R5-tropic viruses are predominant [66, 67], this might also explain the decrease of X4-capable variants in the database over time. The first time period contains also less samples than later time periods, which might influence the trend.

We could detect a trend towards resistance for all 11 bNAbs regarding the subtype B HIV-variants (10/11 if Jonckheere-Terpstra test is used). For the non-B subtype population, we observed the trend for only 6 of the 11 bNAbs (5 of 11 if Jonckheere-Terpstra test is used). A summary of the findings and the corresponding p-values of both statistical tests can be found in S6 Table.

### Conclusion

In this study, we showed that neutralization sensitivity of new HIV-1 variants to broadly neutralizing antibodies (bNAbs) is predictable using neutralization information from existing neutralization assays. The credibility of the models were underlined by the finding that the prediction models learnt important binding sites for the bNAbs implicitly, without explicitly getting this type of information in the learning process. Hence, additional information such as structural binding site information is unlikely to boost the performance significantly. We increased the interpretability of the models, by offering the user more information on the prediction outcome in form of a motif logo where the logo displays the contribution of the pivotal residues of the test sequence to the prediction. In general, our method could be applied as a recommendation tool for bNAbs therapy, but it could already be used in planning clinical trials concerning bNAbs therapy to screen patients before those therapies are approved for clinical use.

It is unquestioned, that an effective bNAb therapy will consist of a combination of bNAbs targeting distinct epitopes on the envelope spike to prevent the emergence of antibody resistance. To determine which and how many bNAbs to choose, several studies analyzed systematically combinations of different bNAbs [68, 69] experimentally but also predicted the neutralization sensitivity using additive models. However, these prediction models need the neutralization sensitivity of the virus to the single bNAbs in the combination as input. Our learnt classifiers could be extended similarly to additive models that predict if or how effective a combination of bNAbs is requiring only the envelope sequence of the virus.

Despite the good performance and biological relevance of our classifiers, the current models are not suited for a direct application in clinical settings. In the clinical setting, it is more tolerable to misclassify a sensitive HIV variant to a bNAb than misclassifying a resistant HIV-1 variant. While the area under the ROC curve was helpful in determining, if the classification task can be accomplished with our proposed methods and for comparison reasons, it is not the best approach to design the models for the final application setting due to the low average specificity for some of the bNAbs (see S5 Table). In order to apply our models in the clinical setting, clinical data has to be analyzed instead of pseudovirus panel data. In addition, an appropriate false discovery rate has to be agreed on with the clinicians, for which the final models can be optimized for. This holds for any method used for this classification task. Apart from their potential use as recommendation tool, computational prediction models can in general be used to analyze the change in the neutralization sensitivity of HIV-1 over time. We could confirm previous results suggesting a trend towards antibody resistance in the subtype B population [55–57]. Moreover, we scaled up the analysis to the global HIV-1 population, showing that there is a general drift towards antibody resistance in the world-wide HIV-1 population for most of the bNAbs. These findings are relevant for the selection of suitable vaccine candidates; a combination of bNAbs is however still very potent in neutralizing HIV-1 [56].

## Materials and methods

### Neutralization assay

We used the IC50 titers of 11 different antibodies (PG9, PG16, 35O22, VRC01, VRC-PG04, 3BNC117, NIH45-46, PGT128, PGT121, 10-996 and 10-1074) for 115 to 220 HIV-1 isolates from three different neutralization assays [10, 26, 47] (see S8 Table). For the bNAbs PG9, PG16, PGT121 and VRC01 neutralization information was available from two neutralization assays. Although the overlap of tested HIV-1 samples was quite high as well as the correlation of the corresponding IC50 titers, we did not merge the information from the two assays for these bNAbs. We represented each HIV-1 isolate by the amino acid sequence of its envelope glycoprotein from the Los Alamos HIV sequence database [48]. We excluded HIV-1 isolates for which no GenbankID was available, or the envelope sequence was shorter than 800 amino acids.

### Data preparation for prediction models

Since the feature vector of each sample has to be of the same length for most of the kernels, we aligned the amino acid sequences with the HIValign tool from the Los Alamos HIV sequence database [48]. For the polynomial and Gaussian RBF kernel the amino acid sequences have to be transformed to a real-valued input. We used one-hot encoding to represent the sequence information for the polynomial kernel, i.e., each amino acid a_i_, i ∈ {1, …20} is transformed into a 20-dimensional vector, where only the *i*-th entry is 1, and the others are 0. For the Gaussian RBF kernel, we encoded the sequence information using physico-chemical properties (RBF1 [70] and RBF2 [71]).

In the classification task, the IC50 titers were converted to -1 if the IC50 value was above 50 μg/mL (resistant), and otherwise to +1 (susceptible) similar to Doria-Rose et al. [47]. Due to their distribution, the IC50 values for the regression task were logarithmized.

### Kernel comparison and parameter settings

To test if *l*-mer string kernels (such as the oligo kernel [52] or the weighted degree kernel with shifts (WDKS) [51]) perform better than conventional kernels (such as the polynomial or the Gaussian RBF kernel), we compared the performances of prediction models based on each of these kernels. The comparison was conducted by 10 runs of a 5-fold nested cross-validation using AUC and Pearson Correlation Coefficient as performance measure for the classification and regression task, respectively. The tested parameter range for each kernel is listed in S1 Table. To determine the best parameter setting for each bNAb prediction model, we performed an additional 5-fold cross-validation. Since in the nested cross-validation mainly small values of the width parameter (2σ^2^) led to high prediction performances, we further sampled the range between 0 and 3 for this parameter.

### Method comparison and parameter settings

We compared the final SVM classifiers based on the oligo kernel with random forests, an SVM using a linear kernel, a neural network, and a logistic regression with lasso regularization (lasso). For the random forest, the neural network, and the lasso approach, the amino acid sequences were mapped to their index in the amino acid alphabet. For the linear kernel, the sequences have been encoded using the one-hot encoding approach, i.e., each amino acid a_i_, i ∈ {1, …20} is transformed into a 20-dimensional vector, where only the *i*-th entry is 1, and the others are 0. While the random forest approach can handle internally categorical variables with more than two factors, we created dummy features for each alignment position for the neural network and the lasso approach. We used the R package randomForest [72] setting the number of variables randomly sampled as candidates at each split (mtry) to the square root of the number of features in the model and the numbers of tree to grow (ntree) to 500. For the neural network, we used the R package neuralnet [73], we used one layer and set the number of hidden layers to the square root of the number of features. To build the logistic regression models, we used the R package glmnet [74] where we used lasso as regularization (*α* = 1) and tuned the lambda parameter in an internal cross-validation. For the linear kernel, we used the R package kernlab [75] setting the kernel to vanilladot using the default cost parameter C. The performance was assessed on 10 runs of stratified 5-fold cross-validation. We did not compare the performance over a nested cross-validation iterating over different hyperparameters for the models, due to the infinite range of possibilities. We used the R package mlr [76] to compare all the methods.

### Retrieving discriminant signals from the oligo kernel

A kernel *k*(*x*, *x*′) can be considered as a similarity function between instances *x* and *x*′. The oligo kernel computes *k*(*x*, *x*′) between two sequences *x* and *x*′ of same length *L* by comparing the co-occurrences of their substrings (*oligomer*) of length *l* with 1 ≤ *l* ≤ *L* within a particular distance (width parameter *σ*^2^). Therefore, the occurrence of a particular *l*-mer in a sequence *x* (denoted as x_ω_) is encoded by the so-called oligo function *μ*

$$\begin{array}{c} \mu_{\omega }\left(t\right)=\sum_{p\in x_{\omega }}\text{exp}\left(-\frac{1}{2\sigma^{2}}{\left(t-p\right)}^{2}\right) \end{array}$$ (1)

with the continuous position variable *t* ∈ [1, *L*] and σ^2^ controlling the positional uncertainty. As described in [52], the corresponding learnt weight of the classifier for each oligomer ωat each position *t* can be retrieved by

$$\begin{array}{c} |w_{\omega }\left(t\right)|=|\sum_{i=1}^{N}\alpha_{i}y_{i}\mu_{\omega }^{i}\left(t\right)|, \end{array}$$ (2)

where *i* ∈ {1, …, *N*} denotes the *i*-th training sample with *α*_*i*_ ≥ 0 and *y*_*i*_ ∈ {−1, 1} being the learnt weight and classification label of the *i*-th sample, and with $\mu_{\omega }^{i}\left(t\right)$ being the oligo function of *l*-mer *ω* of the training sequence *i* at position *t*.

Considering the weights of each oligomer for the test sequence, there exists only one oligomer *ω* containing the actual residue as starting point whose contribution is calculated as

$$\begin{array}{c} S_{\omega }^{*}\left(t\right)=\sum_{i=1}^{N}\alpha_{i}y_{i}<\mu_{\omega }^{i}\left(t\right),\mu_{\omega }^{*}\left(t\right)>, \end{array}$$ (3)

with $\mu_{\omega }^{*}$ being the oligo function of *l*-mer *ω* of the test sequence. For *l*-mers > 1 the computed contribution is assigned to all amino acids of the oligomer. To visualize the motif logos we used Weblogo 3.0 [77].

### Data preparation and analysis of the sequence data for the neutralization sensitivity trend analysis

We used all available envelope sequences from the Los Alamos HIV sequence database [48] (37,137), except the sequences that the prediction models were built on and those that were too short, resulting in 35,524 envelope sequences. For 1586 sequences no date was given, and thus these sequences were excluded as well, resulting in 33,938 considered viral envelope sequences. Before predicting the IC50 value for each test sequence, the sequences were aligned to the data sets using profile-to-profile MUSCLE alignment [78] with the Ugene tool [79]. Instead of predicting the IC50 value, the regression models were trained to predict the logarithmized IC50 value.

To identify a drift towards resistance, we performed a permutation test for umbrella alternatives [58] on the predicted (logarithmized) IC50 values grouped by the six time periods. We applied the umbrella test according to Basso et al. [58, 62] with the provided R code. The umbrella test is a generalization of the Jonckheere-Terpstra test, testing for a peak instead of a monotone trend. A significant peak in the last time period was considered as indicator for an increasing trend in IC50 values and thus, a trend towards bNAb resistance.

### Coreceptor prediction

To predict the coreceptor usage, we used the well established prediction tool geno2pheno[coreceptor] [65]. The prediction tool uses a linear support vector machine to predict whether a sequence is from a X4-capable or an R5-tropic virus, only based on the V3 loop sequence of the viral envelope sequence. For each V3 sequence, geno2pheno[coreceptor] provides the false-positive rate (FPR), which is a measure for the confidence of the prediction. geno2pheno[coreceptor] reports the minimal FPR threshold at which the sequence would be classified as X4-capable. For the manuscript, we used an FPR cutoff of 10% to determine X4-capable (≤ 10%) and R5-tropic viruses (> 10%) as recommended by the European Consensus Group on clinical management of HIV-1 tropism testing [80]. Since there are also reasons for other cutoff choices, we additionally provide the results for the FPR cutoffs according to the German and Austrian treatment guidelines (≤ 5%: X4-capable; ≥ 15%: R5-tropic) in the Supporting Information.

### Coreceptor usage distribution in the Los Alamos HIV sequence database

We used the prediction tool geno2pheno[coreceptor] [65] to determine the coreceptor usage of the 33,938 viral isolates from the Los Alamos HIV sequence database [48]. According to the prediction tool, we excluded in total 545 sequences due to warnings regarding the alignment quality and due to warnings regarding the V3 loop quality (alignment score ≥ 95th percentile).

### Association between coreceptor usage and neutralization by PGT128

For this analysis, we used all available sequences from the CATNAP tool [81], retrieved on 2016-08-10. Since we used a neutralization sensitivity cutoff of 50 μg/mL to determine resistance and susceptibility, all sequences, whose neutralization sensitivity were only given as a cutoff less than 50 μg/mL were excluded. In addition, we excluded two sequences due to poor V3 alignment quality. Coreceptor usage was determined using the prediction tool geno2pheno[coreceptor] [65]. To test whether the sensitivity to an antibody is significantly different with regard to coreceptor usage, we performed a two-sided Fisher’s exact test for the two-by-two contingency tables with resistant and susceptible as the row label and X4-capable/R5-tropic as the column label using significance level = 0.05 with the null hypothesis that there is no difference.

### Implementation details

The prediction and analysis of the neutralization sensitivity were implemented mainly in R [82], version 3.2.1 (2015-06-18) and the R package kernlab [75]. The oligo kernels were computed using a customized version of the Shogun-Toolbox [83] (version 2.0.0). To visualize the motif logos we used Weblogo 3.0 [77].

### Data availability

In S8 Table we provide the virus names that we considered for the prediction models as well as the study ID of the neutralization assay. With this, the corresponding neutralization data can be retrieved from CATNAP [81]. For the trend analysis, we provide the accession numbers of each considered HIV-1 variant in the Los Alamos HIV sequence database [48] in S9 Table.

At https://github.com/annahake/g2p-bnab, we additionally provide the computed kernels for the final models as well as the resampling instance for the 10 runs of stratified 5-fold cross-validation. As mentioned in the Conclusion, the final models are so far not adapted for clinical usage.

## Supporting information

S1 Fig Prediction performance of the regression models. The prediction performance of the 11 SVM regression models based on the oligo kernel was measured by the Pearson correlation coefficient, displayed on the y-axis. The regression models are named according to the bNAb they are trained on, shown on the x-axis. The colors of the boxes refer to the epitope category of the corresponding bNAb. The gray dashed line denotes no linear relationship. The prediction performance was assessed in 10 runs of 5-fold nested cross-validation. Most regression models show good performances (average Pearson correlation coefficient ≥ 0.3).
(TIFF)

S2 Fig Subtype and geographical distribution of HIV-1 variants over the six time periods. For each time period, we display the number of samples from the three most frequent countries as well as the sum of samples from the remaining countries (OTHER). The country distribution is shown for the subtype B (**A**) and the subtype non-B HIV-1 variants (**B**). In **C** we display the number of samples in each time period for the five most frequent subtypes, and additionally the number of samples for the non-B subtypes (dashed line).
(TIFF)

S3 Fig Neutralization sensitivity analysis for the subtype B HIV-1 variants. Predicted neutralization sensitivity of HIV-1 variants (subtype B) from the Los Alamos HIV sequence database to all 11 bNAbs. Neutralization sensitivity (logarithmized IC50 values) was predicted using our SVM regression models based on the oligo kernel. The HIV-1 variants are grouped in six, consecutive, time periods, displayed on the x-axis. A trend towards bNAb resistance was reported if the neutralization sensitivity increased over time with a significant peak in the last time period. The significance was determined using a permutation test for umbrella alternatives and a significance threshold t = α/# total tests = 0.05/22 = 0.0023 with Bonferroni correction for multiple testing.
(TIFF)

S4 Fig Neutralization sensitivity analysis for the non-B subtype HIV-1 variants. Predicted neutralization sensitivity of HIV-1 variants (subtype non-B) from the Los Alamos HIV sequence database to all 11 bNAbs. Neutralization sensitivity (logarithmized IC50 values) was predicted using our SVM regression models based on the oligo kernel. The HIV-1 variants are grouped in six, consecutive, time periods, displayed on the x-axis. A trend towards bNAb resistance was reported if the neutralization sensitivity increased over time with a significant peak in the last time period. The significance was determined using a permutation test for umbrella alternatives and a significance threshold t = α/# total tests = 0.05/22 = 0.0023 with Bonferroni correction for multiple testing.
(TIFF)

S5 Fig Association between coreceptor usage and neutralization sensitivity. For all considered 11 bNAbs, we display the relative number of resistant (orange) and susceptible (blue) strains with respect to their predicted coreceptor usage (R5-tropic or X4-capable). Statistical significance was assessed with a two-sided Fisher’s exact test.
(TIFF)

S6 Fig Prediction performance comparison for different machine learning approaches. For each bNAb classifier, the prediction performance measured by the area under the ROC curve (AUC) is displayed for our SVM models using the oligo kernel, an SVM model using the linear kernel, a logistic regression model with lasso regularization, a random forest model, and a neural network model.
(TIFF)

S1 Table Performance comparison of different kernels and the investigated parameter range. In order to select a kernel for the SVM models, the performance of the polynomial kernel, radial basis function kernel (RBF), weighted degree with shifts kernel (WDKS) and the oligo kernel (Oligo) were compared in 10 runs of a 5-fold nested cross-validation. The cost parameter C of the SVM was sampled in the range from 10E-6 to 10E6 by powers of 10. The two RBF kernels differ in the physico-chemical encoding of the amino acid sequences (see Materials). The parameters of each kernel as well as the sampled range for each parameter are presented in the first sheet. The second sheet contains the prediction performance of each kernel measured by the Area under the ROC curve (AUC) in 10 runs of a 5-fold nested cross-validation exemplarily for all 11 bNAbs. All kernels performed equally well for all bNAbs, apart from VRC-PG04, for which the oligo kernel performed better. Therefore, the oligo kernel was taken to build the prediction models.
(XLSX)

S2 Table Ratio of R5-tropic to X4-capable viruses in the LANL database. The observed percentage of X4-capable and R5-tropic HIV-1 variants in the Los Alamos HIV sequence database over the six considered time-periods. The coreceptor usage was determined using the well-established prediction tool geno2pheno[coreceptor] using an FPR-cutoff of 10% as recommended by the European Consensus Group on clinical management of HIV-1 tropism testing, and the FPR-cutoff recommended by then German and Austrian treatment guidelines (≤ 5%: X4-capable; ≥ 15%: R5-tropic).
(XLSX)

S3 Table Association between coreceptor usage and neutralization by PGT128. HIV-1 variants from a neutralization assay against PGT128 and their coreceptor usage are presented in this contingency table. The coreceptor usage was determined using the well-established prediction tool geno2pheno[coreceptor] using an FPR-cutoff of 10% as recommended by the European Consensus Group on clinical management of HIV-1 tropism testing, and the FPR-cutoff recommended by then German and Austrian treatment guidelines (≤ 5%: X4-capable; ≥ 15%: R5-tropic). To test whether the sensitivity to PGT128 is significantly different with regard to coreceptor usage for each FPR-cutoff, we performed a two-sided Fisher’s exact test using significance level = 0.05.
(XLSX)

S4 Table Performance comparison of the full and reduced prediction models. Performance comparison (AUC) of the full classification models and classification models that were built using the strongest p% beforehand learnt discriminant signals. To compare the performances of the reduced to the full models, we divided our data set into three partitions. Partition A (40% of the data) was used to build a full model (parameters were fitted using a 5-fold cross-validation), from which the p% strongest discriminant signals were extracted. Partition B (40% of the data) was used to build prediction models only based on the p% of the discriminant signals. On the same data set, we also build the full prediction model. The performance of full and reduced models were tested on the third partition C (20% of the data). For each prediction model, we tested the null hypothesis that the full and reduced model have on average the same performance using a paired, two-sided, Wilcoxon test and a significance threshold t = α/#reduced models = 0.05/8 = 0.00625 with Bonferroni correction for multiple testing. For each prediction model, we could not reject the null hypothesis for most of the reduced models. Only five comparisons show a significant p-value (marked in red), which is however very close to the Bonferroni correction threshold.
(XLSX)

S5 Table Prediction performance comparison for different machine learning approaches. For each bNAb classifier, the prediction performance measured by the averaged area under the ROC curve (AUC) is displayed for our SVM models using the oligo kernel, an SVM model using the linear kernel, a logistic regression model with lasso regularization, a random forest model, and a neural network model. The prediction performance was assessed using 10 runs of a stratified 5-fold cross-validation. In addition, we provide the mean sensitivity and mean specificity for each model.
(XLSX)

S6 Table Statistical analysis of the neutralization sensitivity over time. To investigate whether there is a trend towards resistance over time, we performed a Jonckheere-Terpstra test as well a permutation test for umbrella alternatives. The first sheet contains an overview of bNAbs and subtypes for which we were able to detect a trend towards resistance. The second sheet provides the p-values from both tests.
(XLSX)

S7 Table 1% learnt discriminant signals for each bNAb classifier. For each bNAb we display the 1% strongest learnt signals (oligomers), their position compared to the HXB2 reference and the learnt weight. Negative weights denote associations towards resistance, while positive weights denote associations towards sensitivity. The weights for different classifiers are not comparable. Note that a gap signal implies an absence of a certain amino acid at a certain position. Especially if several amino acids are associated with sensitivity or resistance at a certain position, the gap character might be strongly associated with the other direction (resistance or sensitivity), even stronger than the individual amino acid signals.
(XLSX)

S8 Table Neutralization assay data used for the predictions. We built prediction models for each bNAb separately based on existing neutralization assay data. Here, we list for all 11 bNAbs, the considered viruses (virus names) together with the corresponding study ID in CATNAP [81].
(XLSX)

S9 Table Data for the trend analysis. The accession numbers of the HIV-1 envelope amino acid sequences from the Los Alamos HIV sequence database [48] that we used to analyze neutralization sensitivity over time together with the predicted coreceptor usage and our predicted IC50 values.
(XLSX)
