# Supplementary figures and images for: Prediction of HIV-1 sensitivity to broadly neutralizing antibodies shows a trend towards resistance over time
Source: PLoS Comput Biol. 2017 Oct 24;13(10):e1005789. doi: 10.1371/journal.pcbi.1005789 (PMC5669501; doi:10.1371/journal.pcbi.1005789)

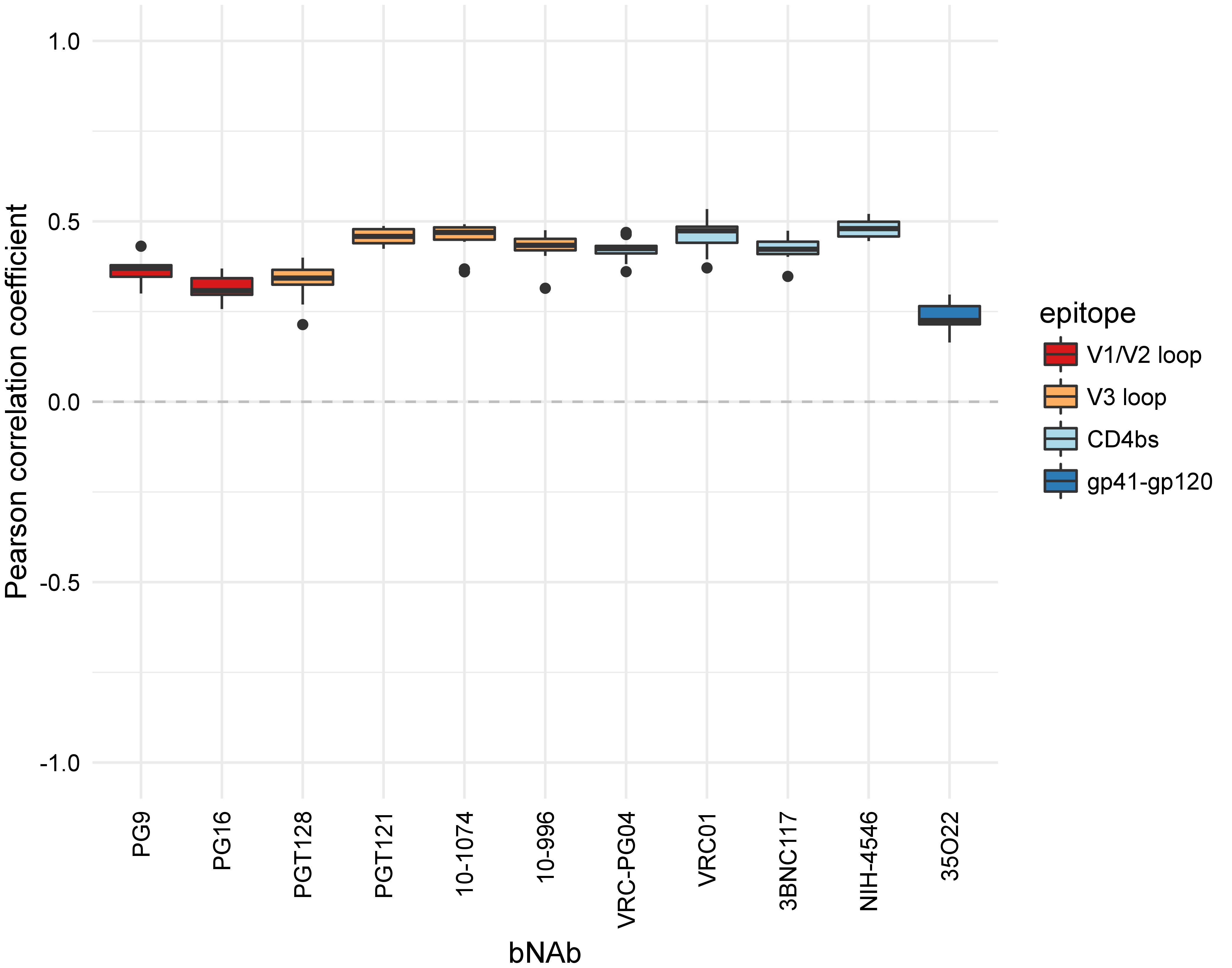

Supplement: S1 Fig — The prediction performance of the 11 SVM regression models based on the oligo kernel was measured by the Pearson correlation coefficient, displayed on the y-axis. The regression models are named according to the bNAb they are trained on, shown on the x-axis. The colors of the boxes refer to the epitope category of the corresponding bNAb. The gray dashed line denotes no linear relationship. The prediction performance was assessed in 10 runs of 5-fold nested cross-validation. Most regression models show good performances (average Pearson correlation coefficient ≥ 0.3). (TIFF) [file pcbi.1005789.s001.tiff]

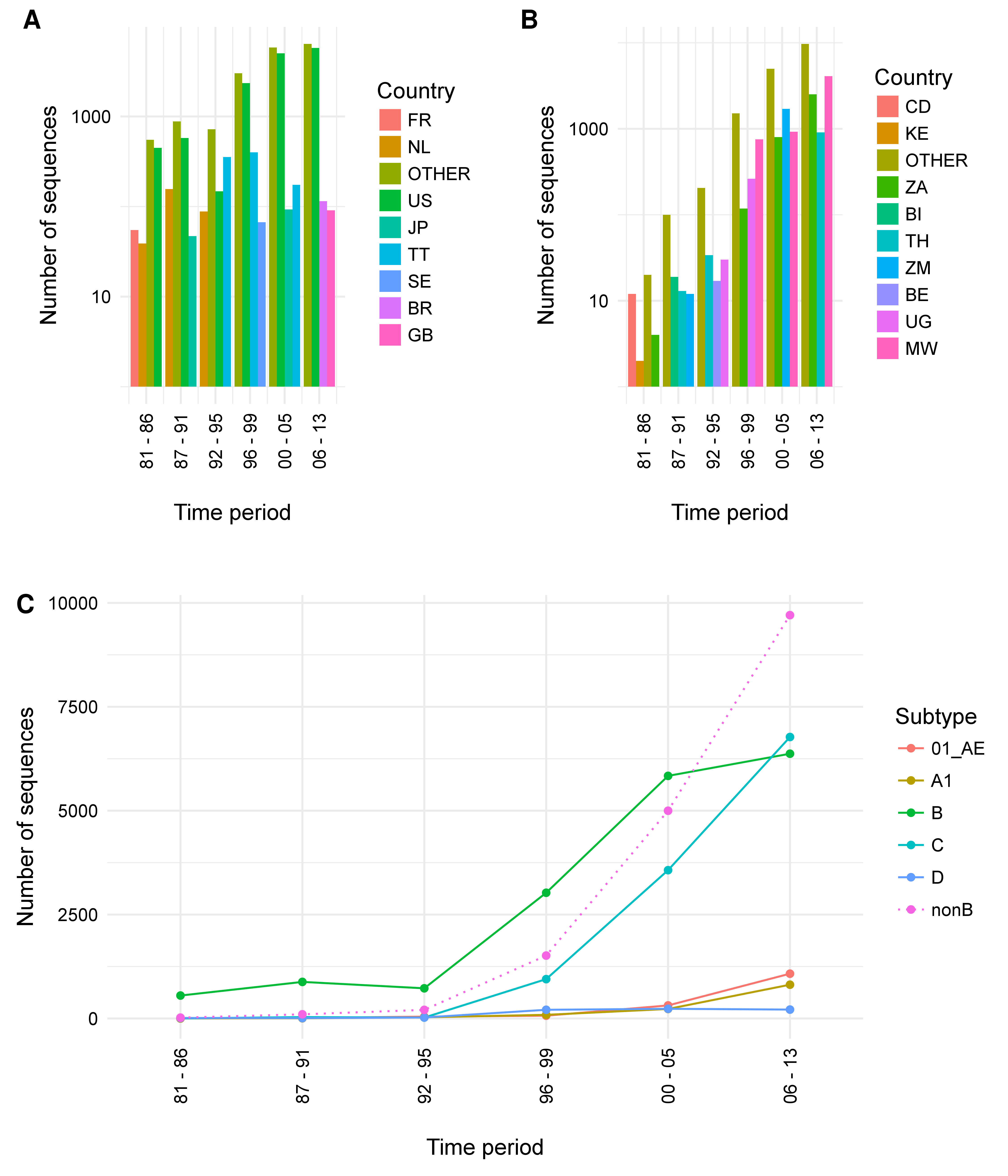

Supplement: S2 Fig — For each time period, we display the number of samples from the three most frequent countries as well as the sum of samples from the remaining countries (OTHER). The country distribution is shown for the subtype B (A) and the subtype non-B HIV-1 variants (B). In C we display the number of samples in each time period for the five most frequent subtypes, and additionally the number of samples for the non-B subtypes (dashed line). (TIFF) [file pcbi.1005789.s002.tiff]

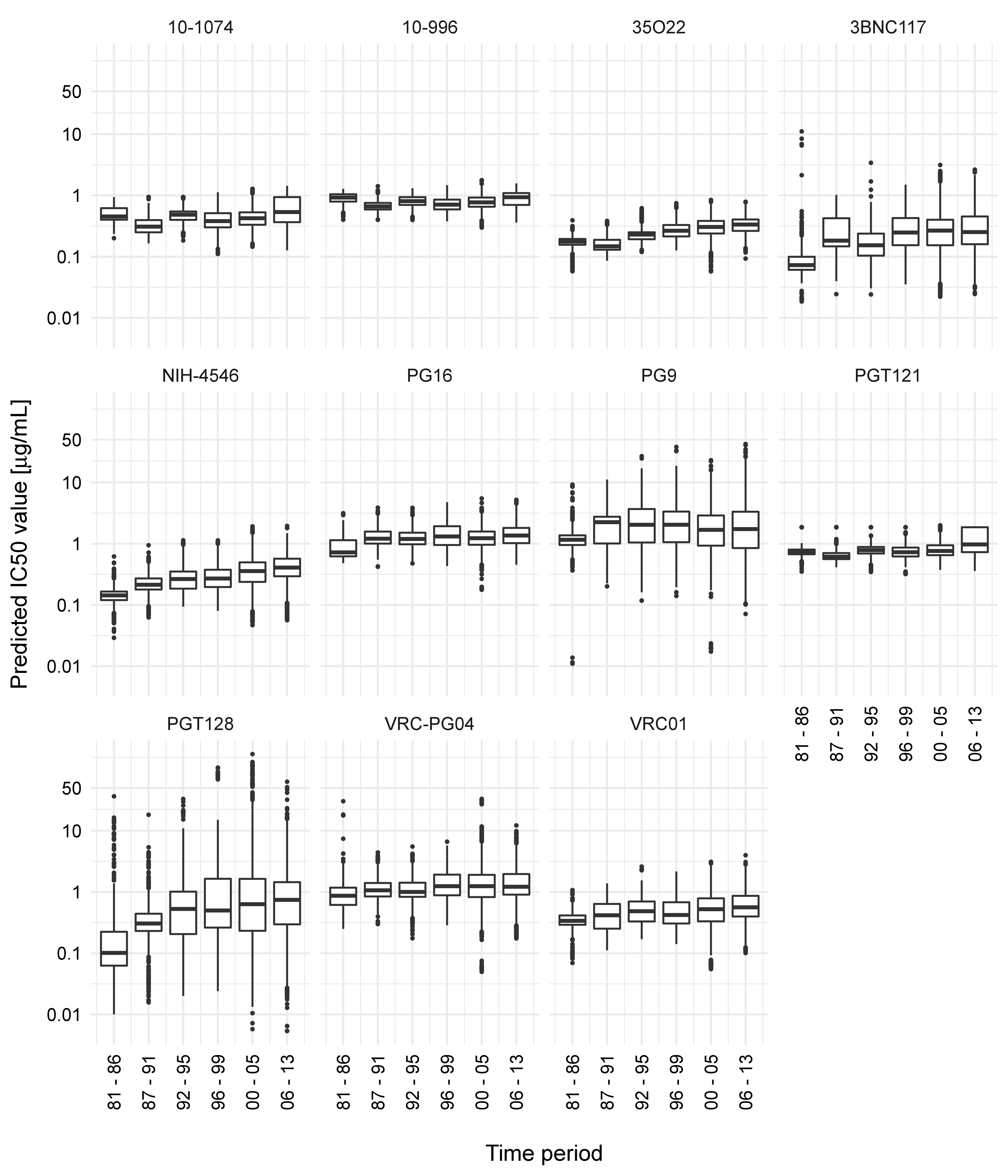

Supplement: S3 Fig — Predicted neutralization sensitivity of HIV-1 variants (subtype B) from the Los Alamos HIV sequence database to all 11 bNAbs. Neutralization sensitivity (logarithmized IC50 values) was predicted using our SVM regression models based on the oligo kernel. The HIV-1 variants are grouped in six, consecutive, time periods, displayed on the x-axis. A trend towards bNAb resistance was reported if the neutralization sensitivity increased over time with a significant peak in the last time period. The significance was determined using a permutation test for umbrella alternatives and a significance threshold t = α/# total tests = 0.05/22 = 0.0023 with Bonferroni correction for multiple testing. (TIFF) [file pcbi.1005789.s003.tiff]

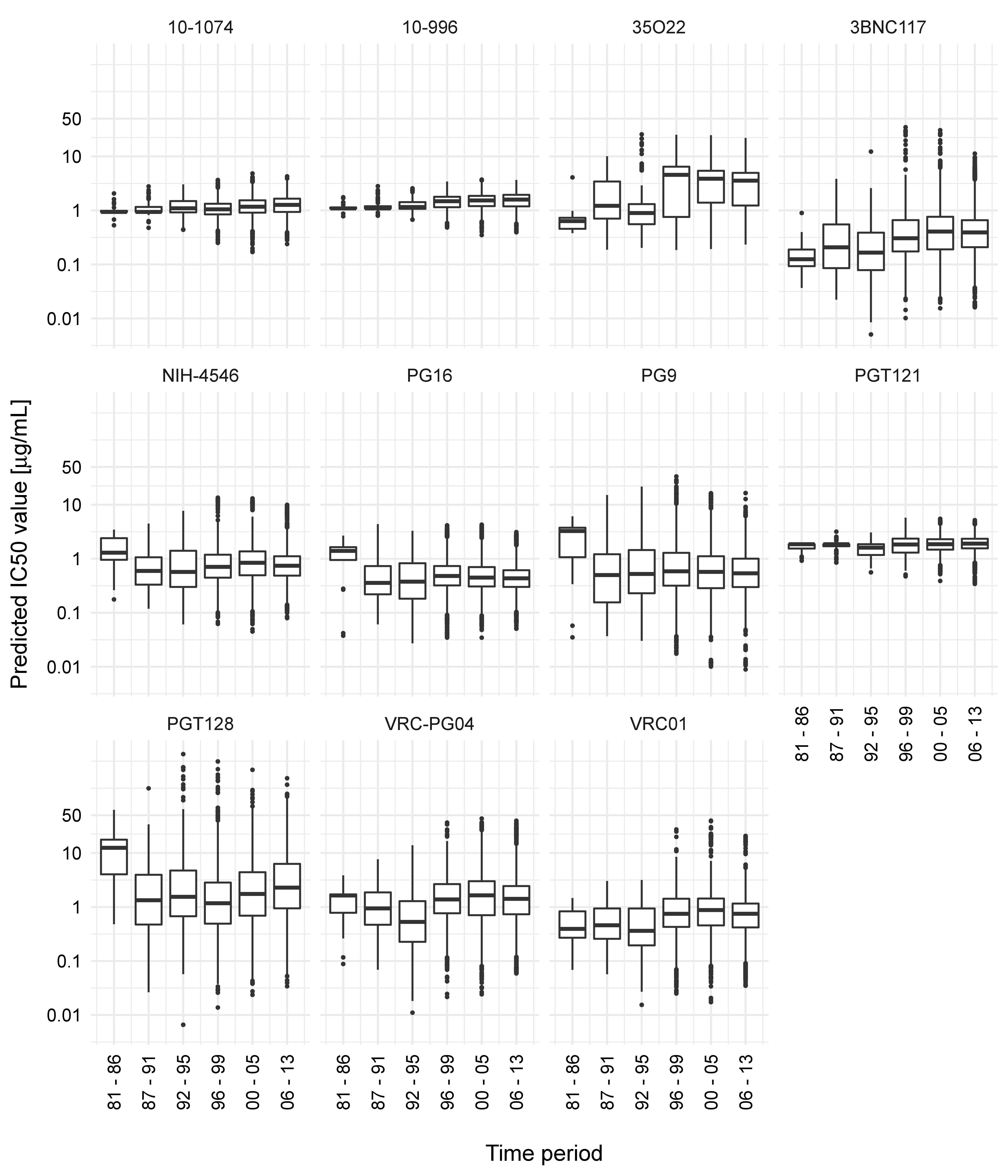

Supplement: S4 Fig — Predicted neutralization sensitivity of HIV-1 variants (subtype non-B) from the Los Alamos HIV sequence database to all 11 bNAbs. Neutralization sensitivity (logarithmized IC50 values) was predicted using our SVM regression models based on the oligo kernel. The HIV-1 variants are grouped in six, consecutive, time periods, displayed on the x-axis. A trend towards bNAb resistance was reported if the neutralization sensitivity increased over time with a significant peak in the last time period. The significance was determined using a permutation test for umbrella alternatives and a significance threshold t = α/# total tests = 0.05/22 = 0.0023 with Bonferroni correction for multiple testing. (TIFF) [file pcbi.1005789.s004.tiff]

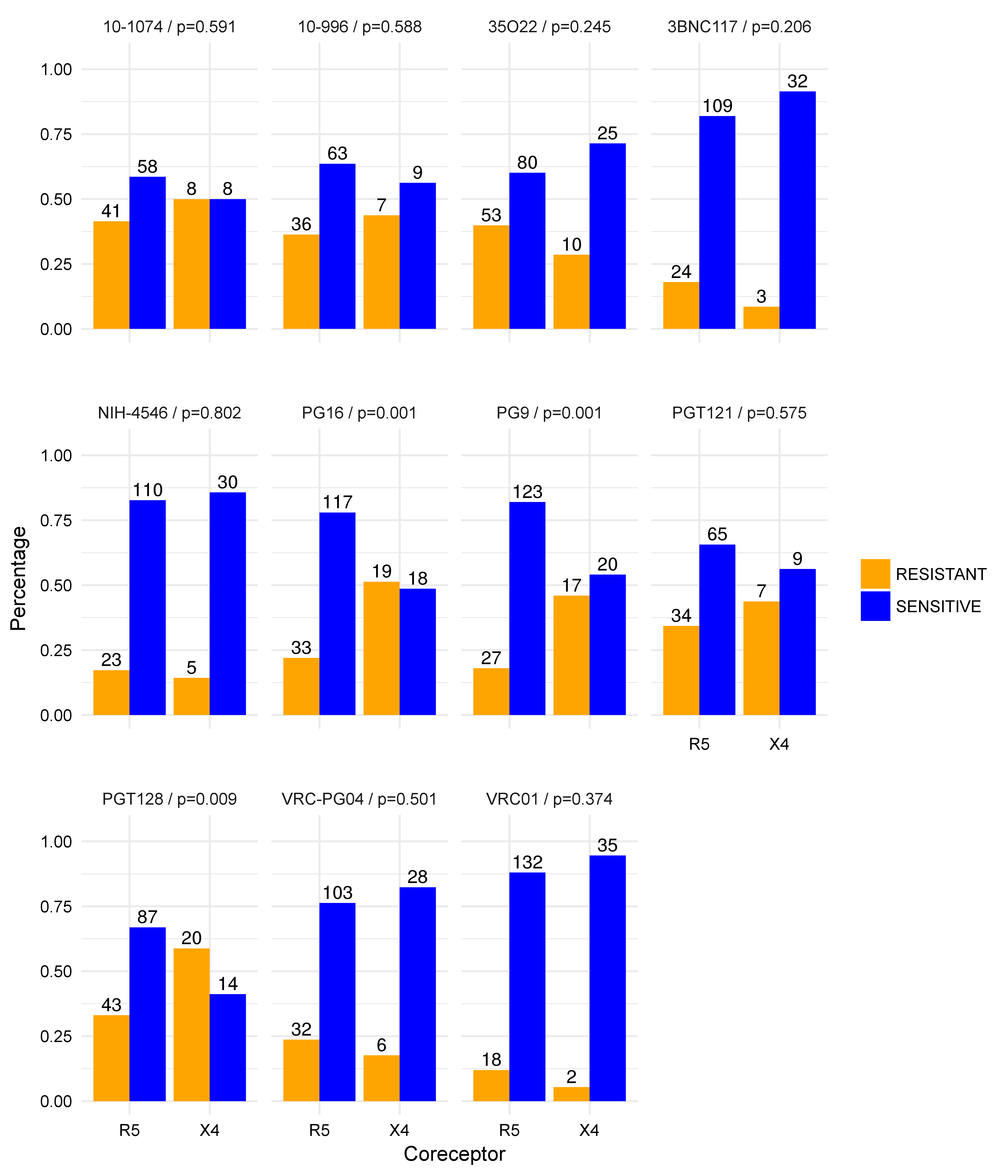

Supplement: S5 Fig — For all considered 11 bNAbs, we display the relative number of resistant (orange) and susceptible (blue) strains with respect to their predicted coreceptor usage (R5-tropic or X4-capable). Statistical significance was assessed with a two-sided Fisher’s exact test. (TIFF) [file pcbi.1005789.s005.tiff]

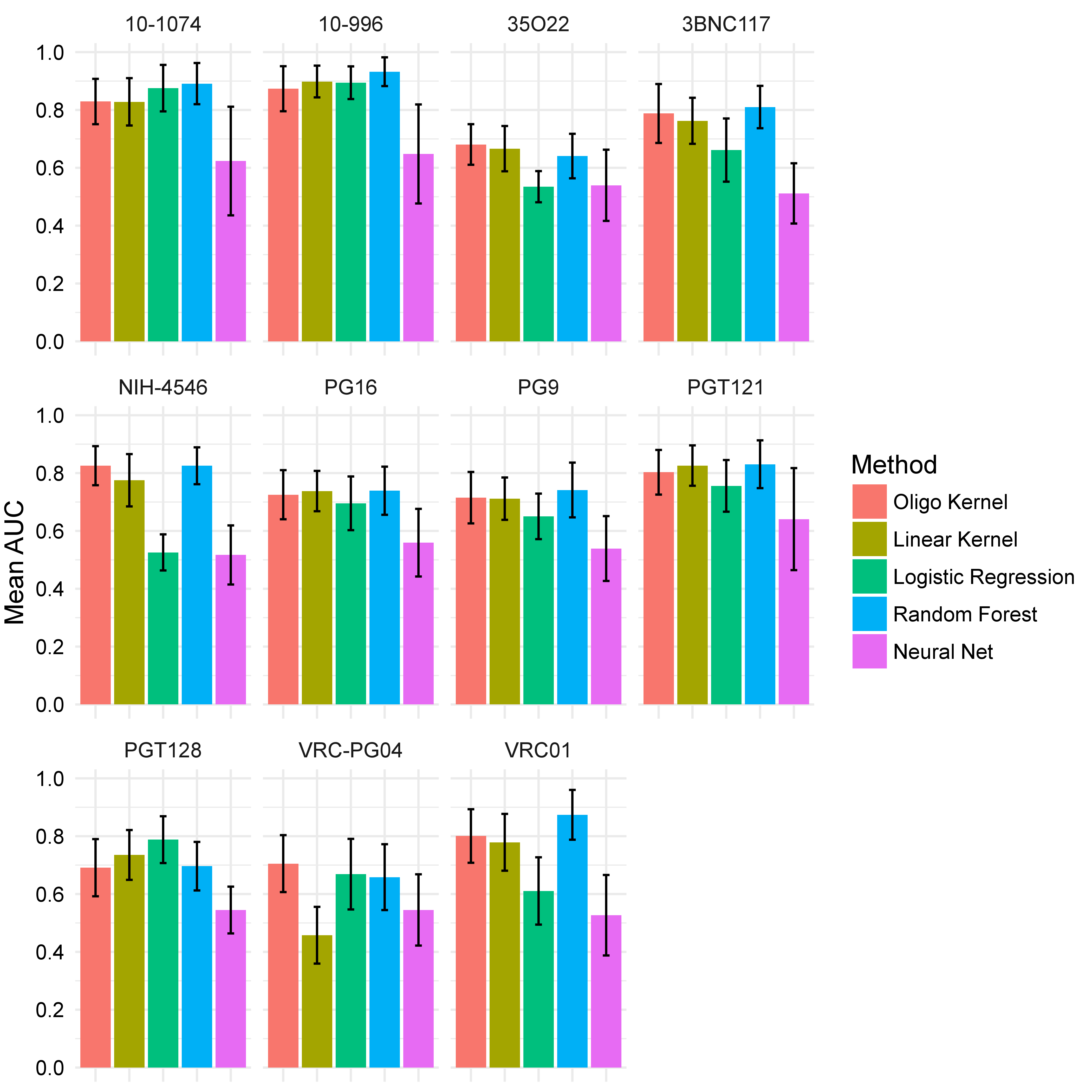

Supplement: S6 Fig — For each bNAb classifier, the prediction performance measured by the area under the ROC curve (AUC) is displayed for our SVM models using the oligo kernel, an SVM model using the linear kernel, a logistic regression model with lasso regularization, a random forest model, and a neural network model. (TIFF) [file pcbi.1005789.s006.tiff]
